# Multiomics landscape of synovial fibroblasts in rheumatoid arthritis

**DOI:** 10.1186/s41232-021-00157-8

**Published:** 2021-03-01

**Authors:** Haruka Tsuchiya, Mineto Ota, Keishi Fujio

**Affiliations:** 1 Department of Allergy and Rheumatology, Graduate School of Medicine, The University of Tokyo, Tokyo, 113-0033 Japan; 2 Department of Functional Genomics and Immunological Diseases, Graduate School of Medicine, The University of Tokyo, Tokyo, 113-0033 Japan

**Keywords:** Rheumatoid arthritis, Synovial fibroblasts, Integrated analysis, Genome, Epigenome, Transcriptome, Genome analysis, Single-cell analysis, DNA methylation, Histone acetylation

## Abstract

**Background:**

Rheumatoid arthritis (RA) is an autoimmune disease characterized by tumor-like hyperplasia and inflammation of the synovium, which causes synovial cell invasion into the bone and cartilage. In RA pathogenesis, various molecules in effector cells (i.e., immune cells and mesenchymal cells) are dysregulated by genetic and environmental factors. Synovial fibroblasts (SFs), the most abundant resident mesenchymal cells in the synovium, are the major local effectors of the destructive joint inflammation and exert their effects through the pathogenic production of molecules such as interleukin-6.

**Main body:**

To date, more than 100 RA susceptibility loci have been identified in genome-wide association studies (GWASs), and finding novel therapeutic targets utilizing genome analysis is considered a promising approach because some candidate causal genes identified by GWASs have previously been established as therapeutic targets. For further exploration of RA-responsible cells and cell type-specific therapeutic targets, integrated analysis (or functional genome analysis) of the genome and intermediate traits (e.g., transcriptome and epigenome) is crucial.

**Conclusion:**

This review builds on the existing knowledge regarding the epigenomic abnormalities in RASFs and discusses the recent advances in single-cell analysis, highlighting the prospects of SFs as targets for safer and more effective therapies against RA.

## Background

**Fig. 1:**
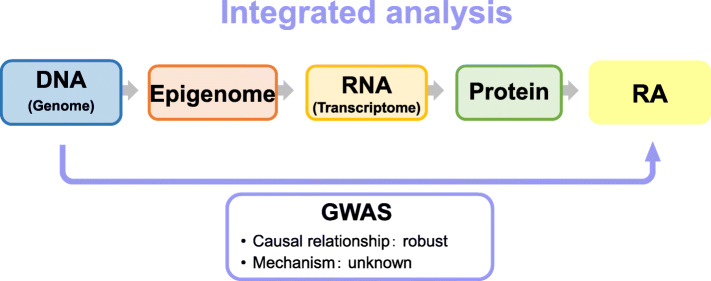
The conceptual diagram of a genome-wide association study and an integrated analysis. GWAS, genome-wide association study

Rheumatoid arthritis (RA) is an autoimmune disease that can severely impair mobility due to persistent synovial inflammation leading to joint destruction. In RA pathogenesis, a variety of molecules in immune cells (e.g., T cells, B cells, and monocytes) and mesenchymal cells are dysregulated under the influence of genetic predisposition and environmental factors. In recent years, genome-wide association studies (GWASs) have identified more than 100 RA susceptibility loci [1]. Although the causal relationship between these “risk single nucleotide polymorphisms (SNPs)” and the final trait “disease” is robust, the biological mechanism that leads to a disease state has not been fully elucidated (Fig. 1). In contrast, genomic studies targeting autoimmune diseases have reported that the majority (> 90%) of risk variants is located in non-coding regions and regulates the expression of several genes in a cell type-specific manner and partly in an environment-specific fashion [2]. To identify the genes and cell types responsible for the pathogenesis of RA, integrated analysis (the so-called functional genome analysis) of the genome and intermediate traits (e.g., transcriptome and epigenome) is crucial.

## RA and synovial fibroblasts

Synovial fibroblasts (SFs) are multifunctional mesenchymal cells of the joint synovium, which are localized in the synovial lining and sublining layers. In the normal synovium, SFs produce substrate proteins (e.g., fibronectin and collagen) and extracellular matrix (ECM)-degrading enzymes (e.g., proteases) to maintain the synovial structure. SFs also contribute to the synovial fluid composition by producing joint lubricants (e.g., hyaluronic acid), and provide nourishment to the underlying articular cartilage.

**Fig. 2:**
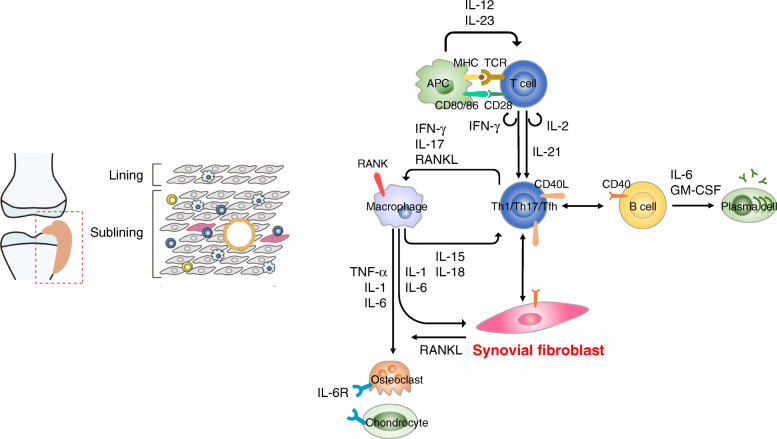
The network of effector cells in the pathology of rheumatoid arthritis. APC, antigen-presenting cell; TCR, T cell receptor; MHC, major histocompatibility complex

On the contrary, in the inflammatory synovium of RA, the lining layer thickens, and immune cells infiltrate the sublining layer. SFs proliferate, and cell-to-cell interactions or humoral factors within the synovium alter SFs into the activated phenotype (Fig. 2). SFs invade and destroy the adjacent articular cartilage, thereby overexpressing adhesion molecules (e.g., vascular cell adhesion molecule-1 (VCAM-1), intercellular adhesion molecule-1 (ICAM-1), and proinflammatory and matrix-degrading mediators (e.g., matrix metalloproteinases (MMPs)) [3]. Particularly, SFs are regarded to be a major source of interleukin (IL)-6, which plays a central role in inflammation and osteoclast activation [4]. Moreover, SFs stimulate vascularization in the synovium through the production of proangiogenic factors (e.g., IL-8 and vascular endothelial growth factor (VEGF)). Angiogenesis promotes the infiltration of immune cells into the synovium and contributes to the persistence of joint inflammation.

## Epigenomic abnormalities revealed in cultured RASFs

To date, knowledge regarding the epigenomic abnormalities related to transcriptional regulation has been accumulated from studies on cultured RASFs. Notably, most conventional studies involve RASFs purified by subculture; hence, the term “culture” has been emphasized.

### DNA methylation

Changes in DNA methylation is the most widely studied epigenetic modification in RASFs. In principle, the expression of genes having CpG islands in the promoter region is suppressed upon methylation by DNA methyltransferase (DNMT). In a study comparing DNA methylation of unstimulated RASFs and SFs from osteoarthritis (OA) patients using methylation arrays, genes in the pathways associated with the pathogenesis of RA (e.g., cell adhesion, cell migration, and interaction between cells and ECM) were found to be specifically hypomethylated in RASFs [5]. It has been indicated that during RA development, epigenomic modifications occur selectively in SFs [6, 7]. Interestingly, differences in DNA methylation of SFs reportedly depend on the RA stage [8, 9]. The recent-onset RA has been found to harbor methylation abnormalities in the gene *Shroom Family Member 1* (*SHROOM1*) that encodes a protein involved in microtubule reconstitution during neural tissue development and cell division. Such genes as *SHROOM1* may therefore constitute biomarkers for the early diagnosis of RA [9]. Furthermore, advanced RA is more intractable to treatment and easily relapses after discontinuation of the drug compared with early RA. Disease relapse could occur from epigenetic modification of SFs caused by the chronic inflammatory environment in the joints. Inflammatory cytokines, represented by tumor necrosis factor (TNF)-α and IL-1β, have been reported to suppress DNMT expression and are involved in DNA demethylation.

### Histone protein acetylation and methylation

Extensive details on abnormalities due to posttranslational modification of histone proteins in RASFs have emerged in recent years. Briefly, when histone proteins are acetylated, DNA binding is loosened to allow the binding of transcription factors, thereby facilitating transcription of the gene. Acetylation is regulated by a balance between histone acetyltransferase (HAT) and histone deacetylase (HDAC). Further, histone protein methylation is induced by histone methyltransferase (HMT). The state of transcriptional activation changes depends on the combination of these modifications and the specific histones or amino acids subjected to the modifications.

Reports state that unstimulated RASFs have transcriptionally active histone modifications (H3K4me3) in the genomic regions encoding MMPs that are directly involved in the degradation of the joints. MMP expression is suppressed by attenuating H3K4 methylation through the knockdown of *WD Repeat Domain 5* (*WDR5*), a component of the HMT complex [10]. Another study reported that the promoter region of *IL6* is subjected to activating histone modification (H3ac) in unstimulated RASFs, and HAT inhibitors suppress the expression of *IL6* [11]. In addition, the promoter region of the *T-Box Transcription Factor 5* (*TBX5*), which regulates the expression of chemokines (e.g., *C-X-C Motif Chemokine Ligand* [*CXCL*] *8*, *CXCL12*, and *C-C Motif Chemokine Ligand 20*), is hypomethylated in RASFs compared with that in OASFs, accompanied by activating histone modifications (H3K4me3 and H3ac) [12]. Loh et al. reported that arthritis-related genes that are persistently expressed in TNF-α stimulate RASFs to show open chromatin structures with activating histone modifications (H3K27ac), and the binding motifs of specific transcription factors (nuclear factor-κB, interferon (IFN) regulatory factors, and activator protein 1) are enriched in their regulatory regions [13].

Some HDAC families are also expected to exert anti-inflammatory effects. For instance, the expression of HDAC5 is suppressed by inflammatory cytokines (e.g., TNF-α and IL-1β), with concurrent induction of the expression of inflammatory mediators such as chemokines, suggesting that HDAC5 plays an anti-inflammatory role [14]. In contrast, inhibition of HDAC3 suppresses the expression of inflammatory mediators induced by IL-1β, indicating a proinflammatory function of HDAC3 [15]. This effect is not observed with HDAC1/2, HDAC6, and HDAC8 inhibition. In other words, there is a functional difference within the HDAC family, and development of RA treatment by selectively inhibiting proinflammatory HDACs is anticipated.

### Recent studies on integrated analysis using cultured RASFs

Several studies have reported the integrated analysis of the transcriptome and epigenome of cultured RASFs. Whitaker et al. identified differentially expressed genes (DEGs) and differentially methylated genes (DMGs) by comparing unstimulated RASFs and OASFs, and compared the DEGs and DMGs with candidate RA susceptibility genes obtained from different databases (cross-sectional analysis) in a GWAS [16]. Among the expected novel therapeutic targets based on these three databases (e.g., *Engulfment And Cell Motility 1* [*ELMO1*], *LBH Regulator Of WNT Signaling Pathway* [*LBH*], and *Protein Tyrosine Phosphatase Non-Receptor Type 11* [*PTPN11*]), the knockdown of *ELMO1*, a protein-coding gene related to cell phagocytosis and movement, suppresses the migration and invasion activities of RASFs. Further, the knockdown of *LBH*, a protein-coding gene involved in embryonic development, increases the growth activity upstream of the *LBH* gene, and the enhancer activity is significantly lower in the risk allele than in the alternative allele. However, the enhancer activity is suppressed by methylation of the enhancer region in either alleles, and therefore, the RA risk SNPs and epigenomic modifications (DNA methylation) are thought to cooperatively control the enhancer activity of *LBH* [17]. *PTPN11*, a gene encoding Src homology region 2 domain-containing phosphatase-2, is highly expressed in RASFs compared with OASFs and is known to be related to the invasion ability of SFs [18]. The enhancer region in which the glucocorticoid receptor-binding motif of *PTPN11* exists is hypermethylated and is involved in enhanced cell sensitivity to glucocorticoid and *PTPN11* expression [19].

Furthermore, Ai et al. built a database consisting of transcriptomic and epigenomic information (histone modifications [H3K27ac, H3K4me1, H3K4me3, H3K36me3, H3K27me3, and H3K9me3], open chromatin, and DNA methylation) of unstimulated RASFs and OASFs. As a result of this multilayer analysis, genes related to Huntington’s disease are reported to be activated in RASFs in addition to known pathways, and invasion of RASFs is found to be suppressed by knockdown of the gene encoding *Huntingtin Interacting Protein 1* (*HIP1*) [20].

### SF subpopulation identification by single-cell analysis of the fresh synovium

The recently developed single-cell RNA sequencing technology has revolutionized SF research. Mizoguchi et al. reported that single-cell RNA sequencing of RA synovium classifies SFs into at least three subpopulations [21]. It has been suggested that CD34^−^THY1^+^ SFs localized around the blood vessels in the sublining represent a pathological subpopulation that produces inflammatory cytokines. Following this study, the Accelerating Medicines Partnership (AMP), which has been active since 2014 at the National Institutes of Health (NIH) in the USA, has conducted single-cell RNA sequencing and mass cytometry of the RA synovium and reported that CD34^−^THY1^+^HLA^−^DR^high^ SFs form a pathological subpopulation that highly expresses IL-6 [4]. Moreover, a study on a mouse model suggested that FAPα^+^THY1^+^ SFs localize to the sublining and are involved in synovial inflammation, while the FAPα^+^THY1^−^ SFs in the lining are involved in bone destruction [22]. Based on these findings, Wei et al. reported that the lining and sublining fibroblasts exist along a gradient that corresponds to the anatomical localization of SFs in the synovium, regulated by endothelium-derived Notch3 signaling [23].

## Prospects

The transcriptomic and epigenomic abnormalities that characterize RASFs have been reported mainly in cultured cells; however, the position of SFs in the formation of inflammatory networks in the synovium has not been fully elucidated. One of the causes is the phenotypic differences between cultured cells and freshly isolated cells. As Wei et al. has clearly described [23], gene expression in SFs, especially induced by signal transduction between heterologous cells, could be homogenized even with two passages or less. In addition, the complexity of the inflammatory environment surrounding SFs complicates the analysis. Within the inflamed joints of RA patients, SFs are thought to acquire pathological traits through exposure to multiple cytokines and direct interaction with immune cells. Some cytokine combinations (TNF-α and IL-17) [24] or costimulatory signals (CD40-CD40L signaling) induce marked cytokine and chemokine expression [25]. Thus, further elucidation of the mechanism of inflammatory amplification of SFs under the influence of cytokine synergy and cell-to-cell communication is warranted. Furthermore, the contribution of the pathological phenotype of SFs at the genomic level (RA risk SNPs) would provide essential information on positioning of SFs in the overall picture of RA. Tsuchiya et al. conducted the first cis-expression quantitative trait locus (cis-eQTL) analysis to identify genetic variants that affect the expression of one or more genes in the activated SFs [25]. This study shed light on the presence of an effect emerging from the stimulatory condition, in addition to the cell type-specific (SFs or immune cells) or disease-specific (RA or OA) eQTL effect. For instance, activated SFs express pathogenic genes, including *CD40* whose induction by IFN-γ is significantly affected by an RA risk SNP (rs6074022). Upon chromatin remodeling in activated SFs, RA risk SNPs have been found to be enriched in clusters of enhancers (super-enhancers; SEs) induced by synergistic proinflammatory cytokines. SEs are large clusters of enhancers collectively bound by an array of transcription factors to define cell identity, and they are hotspots for disease susceptibility [26]. These results suggested that activated SFs play a part in the formation of inflammatory networks associated with RA susceptibility.

As a future development, multi-layer analysis of freshly isolated SFs at the single-cell level (e.g., single-cell QTL analysis integrated with single-cell epigenomics) would be a step forward in understanding the pathophysiology of RA.

## Conclusions

Recent advances in biologics and molecular targeting drugs have led to a paradigm shift in RA treatment. However, maintaining remission with these drugs remains a challenge, and serious adverse events due to systemic immunosuppression often lead to clinical problems. An integrated analytical approach will therefore lead to the development of safer and more effective therapies targeting SFs and provide a breakthrough for next-generation clinical practice.

## Data Availability

Not applicable.

## Abbreviations

cis-eQTL: Cis-expression quantitative trait locus
DEG: Differentially expressed gene
DMG: Differentially methylated gene
DNMT: DNA methyltransferase
ECM: Extracellular matrix
GWAS: Genome-wide association study
HAT: Histone acetyltransferase
HDAC: Histone deacetylase
HMT: Histone methyltransferase
ICAM1: Intercellular adhesion molecule-1
IFN: Interferon
IL: Interleukin
MMP: Matrix metalloproteinase
OA: Osteoarthritis
RA: Rheumatoid arthritis
SE: Super-enhancer
SF: Synovial fibroblast
SNP: Single nucleotide polymorphism
TNF: Tumor necrosis factor
VCAM1: Vascular cell adhesion molecule-1
VEGF: Vascular endothelial growth factor
